# Widespread aberrant functional connectivity throughout the whole brain in obstructive sleep apnea

**DOI:** 10.3389/fnins.2022.920765

**Published:** 2022-08-01

**Authors:** Ailin Hou, Xueming Pang, Xi Zhang, Yanmin Peng, Dongyue Li, He Wang, Quan Zhang, Meng Liang, Feng Gao

**Affiliations:** ^1^College of Precision Instrument and Optoelectronics Engineering, Tianjin University, Tianjin, China; ^2^School of Medical Imaging and Tianjin Key Laboratory of Functional Imaging, Tianjin Medical University, Tianjin, China; ^3^Department of Radiology, Characteristic Medical Center of Chinese People’s Armed Police Force, Tianjin, China; ^4^Tianjin Key Laboratory of Biomedical Detecting Techniques and Instruments, Tianjin University, Tianjin, China

**Keywords:** resting-state functional magnetic resonance imaging, functional connectivity, multivariate pattern analyses, obstructive sleep apnea, machine learning

## Abstract

**Objective:**

Obstructive sleep apnea (OSA) is a sleep-related breathing disorder with high prevalence and is associated with cognitive impairment. Previous neuroimaging studies have reported abnormal brain functional connectivity (FC) in patients with OSA that might contribute to their neurocognitive impairments. However, it is unclear whether patients with OSA have a characteristic pattern of FC changes that can serve as a neuroimaging biomarker for identifying OSA.

**Methods:**

A total of 21 patients with OSA and 21 healthy controls (HCs) were included in this study and scanned using resting-state functional magnetic resonance imaging (fMRI). The automated anatomical labeling (AAL) atlas was used to divide the cerebrum into 90 regions, and FC between each pair of regions was calculated. Univariate analyses were then performed to detect abnormal FCs in patients with OSA compared with controls, and multivariate pattern analyses (MVPAs) were applied to classify between patients with OSA and controls.

**Results:**

The univariate comparisons did not detect any significantly altered FC. However, the MVPA showed a successful classification between patients with OSA and controls with an accuracy of 83.33% (*p* = 0.0001). Furthermore, the selected FCs were associated with nearly all brain regions and widely distributed in the whole brain, both within and between, many resting-state functional networks. Among these selected FCs, 3 were significantly correlated with the apnea-hypopnea index (AHI) and 2 were significantly correlated with the percentage of time with the saturation of oxygen (SaO_2_) below 90% of the total sleep time (%TST < 90%).

**Conclusion:**

There existed widespread abnormal FCs in the whole brain in patients with OSA. This aberrant FC pattern has the potential to serve as a neurological biomarker of OSA, highlighting its importance for understanding the complex neural mechanism underlying OSA and its cognitive impairment.

## Introduction

Obstructive sleep apnea (OSA) is a common sleep-related breathing disorder resulting from obstruction of the upper airway, and the symptoms include snoring at night, frequent stop in breathing, and daytime sleepiness (Park et al., 2011). The major consequences of OSA contain intermittent nocturnal hypoxia and fragmented sleep (Verstraeten, 2007). About 936 million people worldwide between the ages of 30 and 69 years suffered from OSA, when the apnea-hypopnea index (AHI) ≥ 5/h criterion was used (Benjafield et al., 2019). OSA not only increases the risk of hypertension, cardiovascular disease and diabetes, as well as traffic accidents, but also shows an impairment of cognitive functions, such as attention, working memory, episodic memory, and executive function (Gagnon et al., 2014). Moreover, OSA has also been reported to be associated with psychological and neurological problems, such as depression, anxiety, post-traumatic stress disorder, and Alzheimer’s disease (Gupta and Simpson, 2015; Vanek et al., 2020). Sleep fragmentation and intermittent nocturnal hypoxia are considered the main contributing factors to neuropsychological impairments in patients with OSA (Lim and Pack, 2014). However, the neural mechanisms are still largely unclear.

Resting-state functional magnetic resonance imaging (fMRI) provided a non-invasive and effective tool to explore the human brain. Functional connectivity (FC) was a commonly used technique for studying the neural mechanisms underlying cognitive impairment in patients with OSA. In the resting-state FC studies of OSA, researchers found abnormal FCs in patients with OSA associated with several brain regions such as insula (Zhang et al., 2015; Park et al., 2016a), hippocampus (HIP) (Song et al., 2018; Zhou et al., 2020a), amygdala (Yu et al., 2019), caudate nuclei (Song et al., 2018), and posterior cingulate cortex (PCC) (Qin et al., 2020). Besides, a fair amount of studies on OSA reported abnormal within-network and between-network FCs of resting-state brain functional networks (Khazaie et al., 2017), such as default mode network (DMN) (Zhang et al., 2013; Peng et al., 2014; Li et al., 2015, 2016b; Chen et al., 2018a), central executive network (CEN) (Zhang et al., 2013), and salience network (SN) (Yu et al., 2019). However, all these studies on the alterations of resting-state FCs in OSA were based on univariate analysis, i.e., comparing a single FC between patients and controls at a time and repeating this univariate comparison for every FC (i.e., a mass univariate analysis). Therefore, it is unclear whether OSA has a characteristic pattern of FC alterations which can serve as a neuroimaging biomarker for identifying OSA.

Multivariate pattern analysis (MVPA) is a machine learning technique that uses a pattern classifier to identify the specific spatial pattern of brain activities or connectivities in a particular experimental condition or a group of patients (Mur et al., 2009; Pereira et al., 2009). Unlike the mass univariate analysis employed in the above previous studies which only focused on one FC at a time, MVPA performs a joint analysis of all FCs in the whole brain at once and examines their spatial pattern and, thus, has greater power for detecting the differences in FCs between patients with OSA and controls. The higher sensitivity of MVPA also comes from the fact that it naturally avoids multiple comparisons problem and thus corrections for multiple comparisons are usually not needed (Liang et al., 2019). MVPA has been used successfully in detecting abnormal FC patterns and identifying neuroimaging biomarkers in other diseases, such as major depressive disorder (Zhu et al., 2020), schizophrenia (Hua et al., 2020), and social anxiety disorder (Liu et al., 2015). Zhou et al. (2020b) also used this technique based on the spatial pattern of regional homogeneity (ReHo) of resting-state neural activities to distinguish between patients with OSA and HCs. These studies have shown a promising potential of MVPA to identify the characteristic patterns of FC alterations in patients with OSA.

Therefore, in this study, we aimed to characterize the spatial patterns of resting-state FCs in OSA using MVPA and test its potential to serve as a neuroimaging biomarker to aid the diagnosis of OSA. We first performed univariate analyses to compare every FC between patients with OSA and controls, and then performed MVPA, combined with a feature selection procedure, to distinguish patients with OSA from healthy controls (HCs) using the spatial pattern of FCs. To characterize the model-selected FCs that contributed to the successful classification between patients and controls, we further examined the spatial distribution of these selected FCs and their relationship with the known resting-state functional networks. Furthermore, the relationship between MVPA-selected FCs and disease severity of OSA was explored.

## Materials and methods

### Participants

This study included twenty-one male patients with moderate-to-severe OSA and twenty-one male HCs matched for handedness, education, and age. All subjects were recruited from the Sleep Laboratory of the Respiratory Department of Tianjin Medical University General Hospital. The inclusion criteria for patients with OSA included that the AHI was more than 15 times/h. The inclusion criteria for HCs included (1) the AHI < 5 times/h, (2) no history of sleep breathing disorders, (3) no symptoms of nocturnal snoring confirmed by a physician, and (4) male. The exclusion criteria for both patients with OSA and HCs were as follows: (1) Other sleep disorders except OSA, (2) left-handedness, (3) history of severe hypertension, diabetes, respiratory disease, and cardiovascular disease, (4) mental diseases and other neurological conditions, (5) the score of Mini-Mental State Examination (MMSE) less than 24, (6) alcohol or illicit drug abuse or current intake of psychoactive medications, (7) body weight more than 125 kg, and (8) MRI contraindications such as claustrophobia and metallic implants or devices in the body. This study was approved by the local ethics committee and all subjects signed written informed consent.

The clinical manifestations of all patients included nocturnal snoring, irregular breathing, choking in sleep, and daytime sleepiness. None of them received drug therapy, surgery, or continuous positive pressure (CPAP) treatment. All patients underwent nocturnal polysomnography (PSG), and relevant clinical indicators were calculated based on the PSG results. According to the American Academy of Sleep Medicine (AASM) guidelines, apnea was defined as a reduction ≥ 90% in airflow lasting for at least 10 s during sleep and associated with persistent respiratory effort, and hypopnea was defined as a reduction ≥ 30% in airflow lasting for at least 10 s and accompanied by 4% or more oxygen saturation (Redline et al., 2007). The AHI was the average number of apnea and hypopnea that occurred per hour during sleep. The percentage of time with the saturation of oxygen (SaO_2_) less than 90% of the total sleep time (i.e., %TST < 90%) was recorded. The Epworth Sleepiness Scale (ESS) (Johns, 1991), a self-reported questionnaire assessing the severity of daytime sleepiness, was also recorded. The total score in ESS was 24. An ESS score of more than 6, 11, and 16 was defined as sleepiness, excessive sleepiness, and risky sleepiness, respectively (Kapur et al., 2017; Yu et al., 2019). Furthermore, all subjects were also assessed on MMSE, the most commonly used screening scale for cognitive impairment (Folstein et al., 1975). The maximal score of MMSE was 30. A score between 27 and 30 is considered normal, and a score < 27 is considered cognitively impaired.

### Data acquisition

The MR images were acquired using a 3.0 Tesla MRI scanner (Signa HDx, General Electric, Milwaukee, WI, United States) in Tianjin Medical University General Hospital. To reduce head movements and scanner noise, foam pads and earplugs were used, respectively. The resting-state fMRI data were acquired using an echo-planar imaging (EPI). Its sequence parameters were as follows: repetition times (TR) = 2,000 ms, echo time (TE) = 30 ms, flip angle (FA) = 90°, field of view (FOV) = 240 × 240 mm^2^, matrix = 64 × 64, slice thickness = 3 mm, slice gap = 1 mm, and 38 axial slices. Each functional run included 180 volumes. In a single session, subjects were asked to relax without thinking about anything in particular, keep their eyes closed, and stay awake.

### Functional magnetic resonance imaging data preprocessing

The fMRI data preprocessing was performed using Data Processing and Analysis of Brain Imaging (DPABI; Chinese Academy of Sciences, Beijing, China)^1^ (Yan et al., 2016), which is a convenient plug-in software based on Statistical Parametric Mapping (SPM12)^2^ in MATLAB platform. The first 10 volumes were discarded to eliminate the effects of the instability of the machine and the subjects’ inadaptability to the environment in the very beginning of the scan. After slice-timing correction and six-dimensional head motion correction, the remaining 170 images were spatially normalized to the standard Montreal Neurological Institute (MNI) EPI template with a resampling voxel size of 3 × 3 × 3 mm^3^. The effect of linear drift or trends in signal was removed. Then, several sources of spurious variance were regressed out by linear regression, such as 12 head motion parameters, global mean signal, white matter signal, cerebrospinal fluid, and the spike volumes if the frame-wise displacement (FD) exceeded 0.5 mm. A temporal band-pass filtering (0.01 ≤ *f* ≤ 0.08 Hz) was also performed. The head motion (the maximum displacements and maximum spin) of all participants was less than 2 mm and 2°, respectively.

### Anatomical parcellation and construction of brain network

The cerebrum was segmented into 90 regions by the automated anatomical labeling (AAL) template (Tzourio-Mazoyer et al., 2002). The Pearson’s correlation coefficient of the averaged time series between each pair of regions was calculated to define FC, and then a 90 × 90 symmetric correlation matrix was obtained for every participant. A Fisher’s r-to-z transformation of the correlation coefficients was applied to improve the normality of FC values (Liu et al., 2017).

### Univariate analysis

A two-sample *t*-test was used to compare every FC between OSA and HC groups, and the statistical significance for multiple comparisons was determined by three methods, namely, Bonferroni correction (corrected *p* < 0.05), a false discovery rate (FDR, *q* < 0.05), and the network-based statistic (NBS) approach (corrected *p* < 0.05 determined by 10,000 permutations, with a cluster-defining threshold of *p* < 0.001 by two-sample *t*-tests) (Zalesky et al., 2010). Besides, this univariate analysis was performed using the graph theoretical network analysis toolbox (GRETNA)^3^ (Wang et al., 2015).

### Multivariate pattern analysis

The MVPA was performed using the MVPANI toolbox^4^ (Peng et al., 2020) to classify patients with OSA from HCs. Linear support vector machine (SVM) was used to find a hyperplane between patients with OSA and HCs which had a maximal distance to the support vectors on each side. The SVM model was trained and tested using a leave-one-participant-out cross-validation procedure. In each cross-validation, 41 participants were used to train the classifier and the remaining one participant was used to test the trained classifier. In this way, every participant was used once as a test sample, and the classification accuracy was calculated as the percentage of the correctly classified participants over all participants.

#### Feature selection

As the number of features (i.e., the FCs) was far more than the number of subjects, to avoid over-fitting, a feature selection based on the features’ *F* scores was performed during the model training in each cross-validation step using the following procedure as implemented in the MVPANI toolbox: first, in each cross-validation step, an *F* score was calculated for each FC using an *F*-test comparing the two groups of participants (i.e., patients and controls) in the training dataset, and then all FCs were ranked according to their *F* scores; second, only the top *N* percentage of FCs were selected for building the SVM model that was trained using the training samples and then tested using the test sample; third, this feature selection procedure was repeated for all cross-validation steps for a particular percentage *N*. In this study, a series of *N* (from 10% to 100% in steps of 10%; i.e., ten percentages in total) was tested and a classification accuracy was obtained for each *N*. The final classification accuracy was determined by the highest one among the ten accuracies.

#### Permutation test

The statistical significance of the final classification accuracy (against the chance-level accuracy) was determined and corrected for multiple comparisons using a permutation test (*n* = 10,000) as follows. First, in each permutation step, exactly the same MVPA procedure as described earlier was performed, i.e., a linear SVM combined with the same feature selection procedure (i.e., feature selection based on *F* scores with 10 percentages of selected FCs from 10% to 100% in steps of 10%), except that in every cross-validation step, the class labels of the training samples were randomly shuffled; this procedure yielded 10 chance-level classification accuracies and the highest accuracy was taken as the final accuracy of this permutation step. Second, the first step was repeated 10,000 times, yielding 10,000 highest chance-level accuracies of all permutation steps with which a null distribution of chance-level accuracies was formed. Third, the 10 true classification accuracies obtained from the true labels (each corresponding to a feature selection percentage) were compared with this null distribution, resulting in a *p*-value for each true classification accuracy that was calculated as the percentage of chance-level accuracies greater than or equal to the true classification accuracy. The resultant 10 *p*-values corresponded to the family-wise-error (FWE) corrected *p*-values, and the true accuracies were considered statistically significant if *p* < 0.05.

#### Characterization of the multivariate pattern analysis-selected functional connectivities

To characterize the FCs contributing to the “patients vs. controls” classification, we further examined the FCs selected by the above MVPA procedure in two aspects, i.e., the spatial distribution of the selected FCs and the relationships of the selected FCs with the predefined resting-state functional networks. Here, the MVPA-selected FCs included the FCs selected in at least one cross-validation step during the feature selection that led to the highest classification accuracy.

To examine the spatial distribution of the selected FCs, we visually inspected which parts of the brain were involved in these FCs. Furthermore, we also evaluated the importance of the brain regions associated with these FCs by calculating the weighted degrees of each region. This was done for positive weights and negative weights separately by calculating the sum of all positive weights of the FCs associated with a given region and the sum of all negative weights of the FCs associated with a given region, respectively.

As the resting-state functional networks have been reported to play an important role in many sensory and cognitive functions related to OSA (Zhang et al., 2013, 2015; Khazaie et al., 2017; Chen et al., 2018b; Chang et al., 2020) and their disruptions have been indicated in patients with OSA (Zhang et al., 2015; Park et al., 2016b; Wu et al., 2020), we further examined the relationship of the selected FCs with the predefined resting-state functional networks. Specifically, we categorized the 90 brain regions into 7 functional networks, namely, visual network (VN), somatomotor network (SMN), dorsal attention network (DAN), ventral attention network (VAN), limbic system (LS), frontoparietal network (FPN), and DMN, according to Yeo’s parcellation of the cerebral cortex (Yeo et al., 2011). In our results, we merged DAN and VAN into AN (Table 1). The full name of all brain regions is summarized in Supplementary Table 1. According to such categorization of all brain regions, the MVPA-selected FCs were divided into two sets, namely, intra-network FCs (if an FC connects two regions that belong to the same functional network) and inter-network FCs (if an FC connects two regions that belong to different functional networks). The number of intra-network FCs was standardized by the total number of all possible intra-network FCs (i.e., dividing the number of intra-network FCs by the total number of all possible intra-network FCs), and similarly, the number of inter-network FCs was standardized by the total number of all possible inter-network FCs.

**TABLE 1:** Regions belonging to each functional network.

Network	Regions with abbreviation
VN	CAL, CUN, LING, SOG, MOG, IOG, FFG
SMN	PreCG, ROL, PoCG, PCL, HES, STG
AN	SPG, ITG.R, SMA, INS, MCG, SMG, PUT, PAL
FPN	MFG, MFGorb, IFGoper, IFGtri, IPL
LS	SFGorb, OLF, REC, AMYG, CAU, THA, TPOsup, TPOmid
DMN	SFG, SFGmed, SFGmorb, IFGorb, ACG, PCG, HIP, PHG, ANG, PCUN, MTG, ITG.L

#### Correlations between the selected functional connectivities and clinical variables

To investigate the correlation between selected the FCs and the clinical variables, Pearson correlation analyses were performed, and *p* < 0.005 was considered statistically significant.

## Results

### Demographic and clinical indices

There were no significant differences (two-sample *t*-tests, all *p* > 0.05) between patients with OSA and HCs in age, years of education, or MMSE (Table 2). As expected, patients with OSA had a significantly higher score for the body mass index (BMI) (*t* = 3.893, *p* < 0.001), AHI (*t* = 11.762, *p* < 0.001), %TST < 90% (*t* = 3.792, *p* = 0.001), and ESS (*t* = 8.461, *p* < 0.001).

**TABLE 2:** The demographic and clinical characteristics of patients with OSA and healthy controls.

	OSA patients		Healthy controls		*p*-value
			
	Mean	*SD*	Mean	*SD*	
Age (years)	44.05	7.277	40.62	11.404	0.252
Education (years)	13.48	3.092	14.76	2.914	0.173
BMI (kg/m^2^)	29.52	4.231	24.95	3.173	<0.001*
MMSE	29.48	0.814	29.86	0.359	0.057
AHI	54.35	19.97	2.52	1.401	<0.001*
%TST < 90%	18.66	21.10	0.979	2.654	0.001*
ESS	14.67	7.262	1.10	1.136	<0.001*

*Significant difference between OSA and HC, p < 0.05. BMI, body mass index; MMSE, Mini Mental State Examination; AHI, apnea-hypopnea index;%TST < 90%, percentage of total sleep time spent at oxygen saturations less than 90%; ESS, Epworth sleepiness scale. The full name of all brain regions were summarized in Supplementary Table 1.

### Univariate comparisons of functional connectivities between patients with obstructive sleep apnea and healthy controls

The univariate analyses showed that there were no significant changes in FC between patients with OSA and HCs regardless of the method for multiple comparisons’ correction (*p* < 0.05, Bonferroni corrected; *p* < 0.05, NBS corrected; *q* < 0.05, FDR corrected).

### Classification between patients with obstructive sleep apnea and healthy controls

**FIGURE 1:**
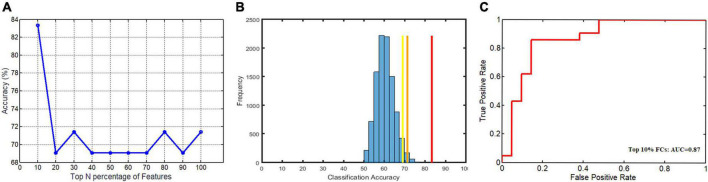
The classification accuracies of 10 feature selection percentages, the corresponding null distribution, and the receiver operating characteristic (ROC) curve for the highest classification accuracies. **(A)** The highest classification accuracy (83.33%) was obtained when the top 10% FCs were selected and two lower accuracies (71.43% and 69.05%) were obtained when different numbers of FCs were selected; **(B)** the corresponding null distribution of the highest chance-level accuracies (the blue histogram), along with the three true classification accuracies (red: 83.33%; orange: 71.43%; yellow: 69.05%); **(C)** the receiver operating characteristic (ROC) curve and the area under the curve (AUC) (0.87) corresponding to this highest classification accuracy (83.33%).

The MVPA showed successful classifications for 4 out of 10 feature selection percentages (Figure 1A): The classification accuracies were 83.33% when the top 10% FCs were selected (*p* = 0.0001; the corresponding specificity and sensitivity were 85.71% and 80.95%, respectively), 71.43% when the top 30%, 80%, and 100% FCs were selected (*p* = 0.0076; the corresponding specificity and sensitivity were 66.67% and 76.19%, respectively), and 69.05% for the other percentages (*p* = 0.067; the corresponding specificity and sensitivity were 71.43% and 66.67%, respectively) (Figure 1B). The highest classification accuracy of 83.33% was considered the final accuracy and the selected top 10% FCs (400 FCs) were further characterized. The receiver operating characteristic (ROC) curve corresponding to this highest classification accuracy (83.33%) and the corresponding area under the curve (AUC = 0.87) are shown in Figure 1C.

### Characterization of the multivariate pattern analysis-selected functional connectivities

**FIGURE 2:**
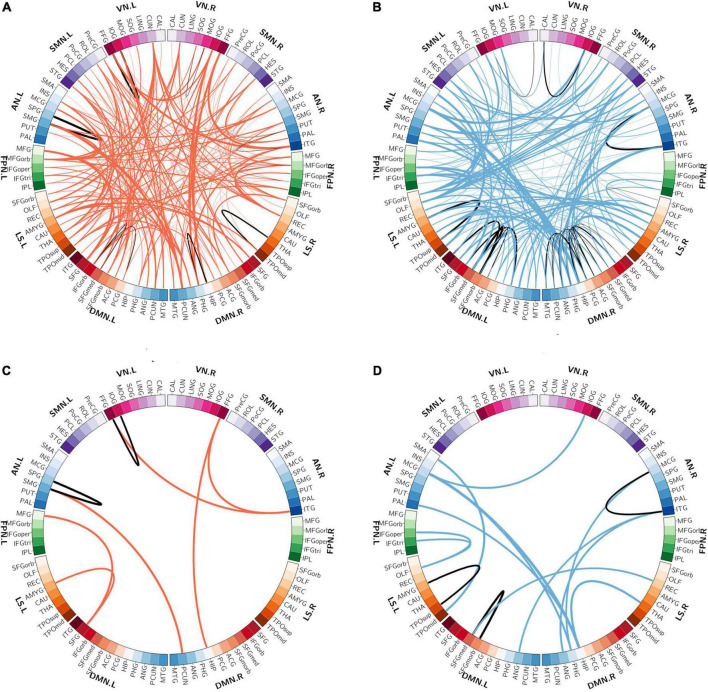
The spatial distribution of the selected functional connectivity. **(A)** The spatial distribution of the selected FCs with higher classification weights in patients with OSA compared with controls. **(B)** The spatial distribution of the selected FCs with lower classification weights in patients with OSA compared with controls. **(C)** The FCs with higher positive weight values were greater than mean + 2SD in patients with OSA. **(D)** The FCs with more negative weight values, which means the absolute weight values were more than mean + 2SD in patients with OSA. The thickness of lines represents the absolute weight values and the black lines indicated functional connectivity within one functional network.SD, standard deviation; VN, visual network; SMN, somatomotor network; AN, attention network; FPN, frontoparietal network; DMN, default mode network; LS, limbic system.

**FIGURE 3:**
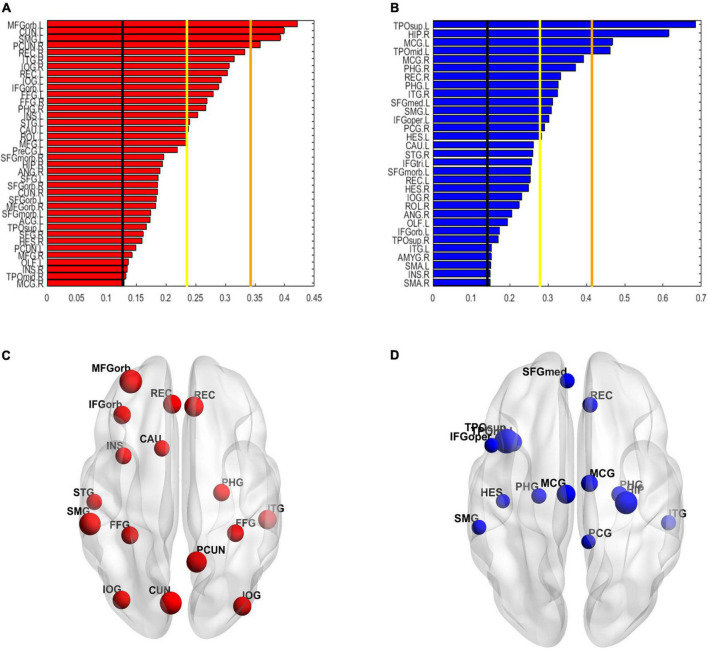
The weighted degree maps of brain regions. **(A,C)** The brain regions with positive weighted degrees greater than mean + SD. **(B,D)** The brain regions with negative weighted degrees less than mean-SD. The black line, yellow line, and orange line indicate the mean value, mean ± – SD, and mean ± – 2SD, respectively. SD, standard deviation.

The spatial distribution of the selected top 10% FCs (i.e., 400 FCs) in the brain is shown in Figure 2. Among the 400 selected FCs, 195 FCs showed higher weight value in patients with OSA (Figure 2A), and 205 FCs showed lower weight value in patients with OSA (Figure 2B). Moreover, the FCs with absolute weight values greater than the mean plus twice the standard deviation (i.e., absolute mean + 2SD) are shown in Figures 2C,D. To evaluate the importance of the brain regions for the classification, we also calculated the positive and negative weighted degrees of each region. A total of 16 brain regions showed significantly higher positive weighted degrees in patients with OSA than in HC (> mean + SD), including the left MFGorb, left CUN, left SMG, right PCUN, right REC, right ITG, right IOG, left REC, left IOG, left IFGorb, left FFG, right FFG, right PHG, left INS, left STG, and left CAU (Figures 3A,C), and 14 brain regions showed significantly lower negative weighted degrees in patients with OSA than in HC, including left TPOsup, right HIP, left MCG, left TPOmid, right MCG, right PHG, right REC, left PHG, right ITG, left SFGmed, left SMG, left IFGoper, right PCG, and left HEC (Figures 3B,D).

**FIGURE 4:**
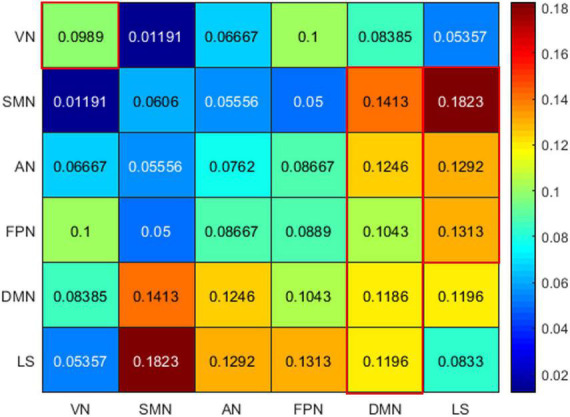
The percentages of intra-network FCs in each functional network (the diagonal entries) and the percentages of inter-network FCs (the off-diagonal entries). The values that exceeded the average percentages were marked with red rectangular boxes. VN, visual network; SMN, somatomotor network; AN, attention network; FPN, frontoparietal network; DMN, default mode network; LS, limbic system.

**FIGURE 5:**
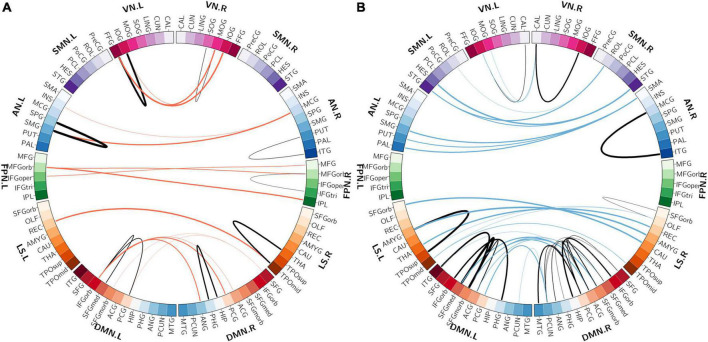
The distributions of intra-network FCs. **(A)** The spatial distribution of the selected intra-network FCs with higher classification weights in patients with OSA compared with controls. **(B)** The spatial distribution of the selected intra-network FCs with lower classification weights in patients with OSA compared with controls. The thickness of lines represents the absolute weight values and the black lines indicate functional connectivity within one functional network. VN, visual network; SMN, somatomotor network; AN, attention network; FPN, frontoparietal network; DMN, default mode network; LS, limbic system.

**FIGURE 6:**
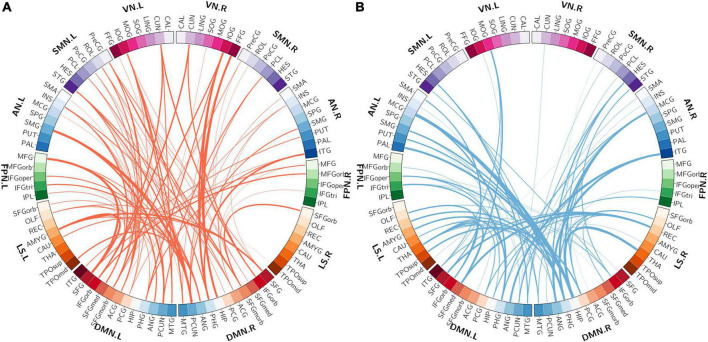
The distribution of inter-network FCs between the DMN and the other five functional networks. **(A)** The spatial distribution of the selected inter-network FCs between DMN and the other networks with higher classification weights in patients with OSA compared with controls. **(B)** The spatial distribution of the selected inter-network FCs between DMN and the other networks with lower classification weights in patients with OSA compared with controls. The thickness of lines represents the absolute weight values. VN, visual network; SMN, somatomotor network; AN, attention network; FPN, frontoparietal network; DMN, default mode network; LS, limbic system.

Among the 400 selected FCs, 65 FCs were intra-network FCs and 335 were inter-network FCs. The numbers of intra-network FCs were 9 in VN, 4 in SMN, 8 in AN, 4 in FPN, 30 in DMN, and 10 in LS, respectively (Figure 4, the diagonal entries; Figure 5), accounting for 9.89%, 6.06%, 7.62%, 8.89%, 11.86%, and 8.33% of intra-network FCs in these functional networks, respectively (the mean percentage of intra-network FC was 9.56% across these functional networks). The percentages of inter-network FCs between each pair of functional networks are shown in Figure 4 (the off-diagonal entries), and the mean percentage was 10.08%. To specifically look at the inter-network FCs associated with the DMN, we also showed all inter-network FCs between DMN and the other five networks in Figure 6.

### Correlations between the selected functional connectivities and clinical variables

**FIGURE 7:**
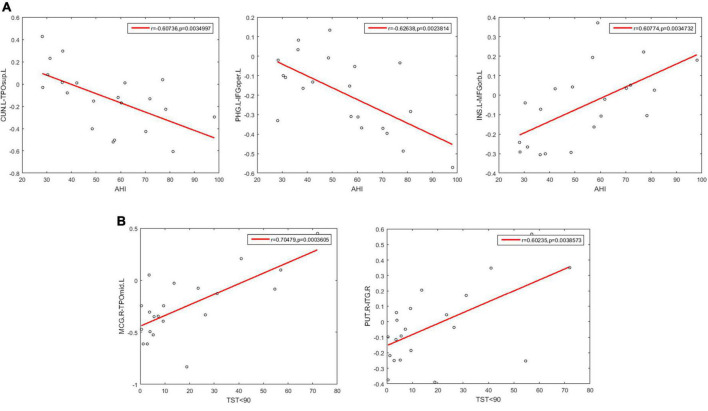
Scatter plots showing significant correlations between the FCs and the clinical variables in patients with OSA. **(A)** The correlations between the FCs and AHI. **(B)** The correlations between the FCs and % TST < 90%. AHI, apnea-hypopnea index; %TST < 90%, percentage of total sleep time spent at oxygen saturations less than 90%; CUN.L, left Cuneus; TPOsup.L, left Temporal pole: superior temporal gyrus; PHG.L, left Parahippocampal gyrus; IFGoper.L, left Inferior frontal gyrus, opercular part; INS.L, left Insula; MFGorb.L, left Middle frontal gyrus, orbital part; MOG.R, right Middle occipital gyrus; TPOmid.L, left Temporal pole: middle temporal gyrus; PUT.R, right Lenticular nucleus, putamen; ITG.R, right Inferior temporal gyrus.

We further examined the correlations between the 400 selected FCs and the clinical variables in patients with OSA. We found that the clinical variable AHI showed negative correlations with the FC between the left CUN and the left TPOsup (*r* = −0.607, *p* = 0.0035) and with the FC between the left PHG and the left IFGoper (*r* = −0.6.26, *p* = 0.0024) and showed positive correlations with the FC between left INS and left MFGorb (*r* = 0.608, *p* = 0.0035) (Figure 7A). Moreover, the clinical variable %TST < 90% showed a positive correlation with the FCs between the right MCG and the left TPOmid (*r* = 0.705, *p* = 0.00036) and with the FC between the right PUT and ITG (*r* = 0.602, *p* = 0.00386) (Figure 7B).

## Discussion

### Weak alterations of resting-state functional connectivities in obstructive sleep apnea can be detected by multivariate pattern analysis

We performed both a univariate analysis and an MVPA to identify the differences in resting-state FCs between patients with OSA and HCs. The fact that no significant differences were identified using univariate two-sample *t*-tests, even for relatively liberal thresholds (*q* < 0.05 corrected by FDR, or *p* < 0.05 corrected by NBS), suggests that the alterations of the resting-state FCs might not be very large. However, such weak alterations of FCs in OSA can be detected as a pattern change by MVPA, confirming that the resting-state FCs were indeed altered in patients with OSA. A classification accuracy of 83.33% also suggests that the spatial pattern of resting-state FCs can successfully distinguish patients with OSA from HCs, demonstrating its potential as a neuroimaging biomarker for aiding the diagnosis of OSA.

### The whole-brain resting-state functional connectivities were altered in a dispersed way in obstructive sleep apnea

Using an SVM classification algorithm combined with a feature selection procedure, we identified 400 FCs contributing to the successful classification between patients with OSA and HCs that yielded the highest classification accuracy (83.33%). Further examination of the spatial distribution of these 400 MVPA-selected FCs showed that almost all brain regions (88 out of 90) were involved in these FCs. This result suggests that OSA is likely to affect the FCs among widely distributed regions in the whole brain, rather than some local networks involving only a few particular brain regions. This is in line with previous studies investigating the FC changes in OSA. For example, Park et al. found 27 decreased FCs and 46 increased FCs in patients with OSA associated with 62 out of 90 brain regions (Park et al., 2016b). Such widely distributed FC alterations also support the previous findings that the global topological properties of the whole-brain resting-state functional network were disrupted as well in patients with OSA. For example, although a small-world topology was still preserved, the small-world property was significantly decreased (Chen et al., 2017), along with some other global topological properties such as clustering coefficient, characteristic path length, and global efficiency (Huang et al., 2019).

### The relationship of the multivariate pattern analysis-selected functional connectivities with the predefined resting-state functional networks

We also characterized the relationship of the MVPA-selected FCs with the predefined resting-state functional networks and found that these FCs were associated with all 7 predefined resting-state functional networks, further corroborating the finding that OSA involves widely distributed FC alterations in the whole brain. Furthermore, the quantification of these MVPA-selected FCs in terms of intra- and inter-network FCs showed that the DMN had the highest percentage of intra-network FCs among the 400 MVPA-selected FCs and also had relatively high percentages of inter-network FCs with the SMN, the AN, and the LS, suggesting the important role of the DMN-associated FC changes in OSA. This is consistent with some previous studies that have reported abnormal intra-network FCs as well as the global and local topological properties of DMN in patients with OSA compared with HCs (Zhang et al., 2013; Peng et al., 2014; Li et al., 2015, 2016a,b; Chen et al., 2018a). It is known that the DMN, including the posterior cingulate gyrus (PCG), the medial prefrontal cortex, HIP, medial temporal lobe (MTG), angular gyrus (ANG), and precuneus (PCUN) as the core regions (Li et al., 2016b), is more active during resting state but its activity is inhibited during many cognitive tasks, and the degree of inhibition even increases with the task load (Buckner et al., 2008). Our results showed positive classification weights for the FCs between the bilateral PCUN, the bilateral PCG, the right SFG, and the medial part of the right superior frontal gyrus (SFGmed), indicating that these FCs were lower in patients with OSA compared with HCs. The connectivity between the right HIP and ipsilateral parahippocampal gyrus (PHG) was an important member of the classified pattern, while Song’s study found reduced FC between the right HIP and the bilateral thalamus and PHG in patients with OSA (Song et al., 2018). We also found that the intra-network FC in DMN associated with bilateral PCG was useful in OSA-HC classification. PCG, PCUN, and HIP were considered the key regions of posterior DMN (pDMN); the medial prefrontal cortex, anterior cingulate (ACG), and superior frontal gyrus belong to anterior DMN (aDMN) (Zhang et al., 2013; Chen et al., 2018a). In a previous study, Zhang et al. (2013) found that FCs of patients with OSA in aDMN were significantly decreased than HC’s, while FCs in OSA were increased in pDMN. We also found that the intra-network FCs in aDMN showed negative classification weights, such as the FCs between the bilateral ACG and the bilateral medial orbital part of the superior frontal gyrus (SFGmorb), Therefore, the abnormal intra-network FC in DMN explained the functional heterogeneity of aDMN and pDMN. Chen et al. (2018a) reported abnormal FCs within the DMN and decreased network topological properties such as the clustering coefficient and the local efficiency of the DMN.

Moreover, some previous studies have reported abnormal FC between DMN and other brain regions. Zhang et al. (2015) found that the FCs between key nodes of the DMN (the bilateral ACG, right PCG, bilateral SFG, and bilateral medial prefrontal cortex) and the AN (the right INS) were significantly decreased in patients with OSA. Song et al. (2018) reported that the FCs between the nodes in DMN (HIP and ANG) and the nodes in LS (THA and CAU) were significantly abnormal in patients with OSA. As the DMN has been suggested to play an important role in many cognitive functions such as regulating emotion, consciousness, memory, and introspection, our present findings, together with the previous results, suggest that the disrupted FCs associated with the DMN in OSA may underlie the cognitive impairments observed in patients with OSA.

Some papers have also found visual dysfunction in patients with OSA. Giora et al. (2017) found that the reaction time in a visual task for patients with OSA was significantly longer than HCs. Moghimi et al. (2013) detected that the nerve fiber indicator was significantly reduced in patients with OSA, and patients with OSA had a higher prevalence rate of glaucoma and ocular hypertension. The calcarine cortex (CAL) is a core region of the visual recognition network (Tao et al., 2013) and was reported to be associated with the shifting of attention to the intended visual target and the modulation of visual input through attention. Liu et al. (2017) found the voxel mirrored homotopic connectivity (VMHC) in bilateral CAL, and VMHC value in CAL was positively correlated with AHI (Yamagishi et al., 2005). Zhang et al. (2013) found that the right cuneus (CUN) exhibited reduced gray matter volume (GMV) in patients with OSA that imply the visual attention deficit of OSA. However, there are few studies on functional disconnection associated with VN in patients with OSA. In the current OSA-HC classification FC pattern, the percentage of intra-network FC in VN was higher than the percentage of intra-network FC in the whole brain, but the percentage of inter-network FC between VN and the other network was lower than the percentage of inter-network in the whole brain. Furthermore, the FC between left CAL and left IOG, FC between right CAL and bilateral IOG, and left MOG play an important role in differentiating patients with OSA and HCs, and the classification weight of these connectivities was negative (HC-OSA).

### Correlations between the functional connectivities and the disease severity

We found that some MVPA-selected FCs were significantly correlated with the AHI and the%TST < 90%. AHI is the main indicator of the severity of OSA. In this study, the FCs between the left PHG (DMN) and left IFGoper (FPN) and between the left CUN (VN) and left TOPsup (LS) showed negative correlations with AHI, while the FC between the left INS (AN) and left MFGorb (FPN) showed a positive correlation of AHI. Although this has not been reported in previous studies, our result suggests these FCs might be indicative of the AHI in OSA.

The FCs between the right MCG (AN) and the left TPOmid (LS), and between the right PUT (AN) and the right ITG (AN) showed a positive correlation with % TST < 90%. It is noticeable that both FCs were associated with the functional network AN (attention network). Attention is a primary cognitive function, involving selective attention, sustained attention, and attention distribution, which will further affect other cognitive functions (Olaithe et al., 2018), and some previous studies have reported attentional impairments in all three aspects of attention in patients with OSA (Verstraeten et al., 2004; Vanek et al., 2020). Even though the treatment of continuous positive airway pressure (CPAP) could improve alertness and attention (Verstraeten and Cluydts, 2004), it did not seem to be able to restore the quality of attention to normal levels in patients with OSA (Lau et al., 2010). Our results provide evidence for the neural mechanisms of attention impairment in patients with OSA, which may be related to the disrupted FCs in the AN due to hypoxemia during sleep, and such attention deficits in patients with OSA may be more resistant to treatment.

### Limitations

There are several limitations in this study. First, the sample size in this study was relatively small and only recruited male subjects. The statistical significance of the correlations between the FCs and the clinical variables was not corrected for multiple comparisons also due to the small sample size. Therefore, large sample dataset and female patients with OSA should be included in future studies to confirm our results. Second, we only used FC to distinguish patients with OSA from HCs. Whether merging different imaging measures could improve the classification accuracy in distinguishing patients with OSA from HCs needs to be further studied.

## Conclusion

The findings in this study revealed that the resting-state FCs were altered in OSA and the disrupted FCs were widely distributed and involved almost all resting-state functional networks in the whole brain of patients with OSA. The successful classification between patients with OSA and HCs obtained using machine learning techniques also indicates that the altered resting-state FCs are indicative of the severity of the disease and have the potential to serve as a neuroimaging biomarker of OSA.

## Data availability statement

The raw data supporting the conclusions of this article will be made available by the authors, without undue reservation.

## Ethics statement

The studies involving human participants were reviewed and approved by the Medical Research Ethics Committee of Tianjin Medical University General Hospital. The patients/participants provided their written informed consent to participate in this study.

## Author contributions

AH and ML: study design and article writing. QZ: acquisition of data. AH, XP, and HW: analysis and interpretation of data. XZ, YP, and DL: technical guidance. ML, QZ, and FG: manuscript review and editing. ML and FG: supervision. All authors contributed to the article and approved the submitted version.

## Footnotes

1
http://rfmri.org/DPABI

2
http://www.fil.ion.ucl.ac.uk/spm

3
http://www.nitrc.org/projects/gretna/

4
http://funi.tmu.edu.cn
