# Supplementary material for: Widespread aberrant functional connectivity throughout the whole brain in obstructive sleep apnea
Source: Front Neurosci. 2022 Aug 1;16:920765. doi: 10.3389/fnins.2022.920765 (PMC9377518; doi:10.3389/fnins.2022.920765)
Supplement: Supplementary file 1 [file Table_1.DOCX]

Supplementary Material

# Supplementary Tables

**Table S1.** The full name of 90 brain regions of automated anatomical labeling template (AAL).

| **Labels** | **Regions** | **Abbreviation** | **Side** |
| --- | --- | --- | --- |
| 1  2  3  4  5  6  7  8  9  10  11  12  13  14  15  16  17  18  19  20  21  22  23  24  25  26  27  28  29  30  31  32  33  34  35  36  37  38  39  40  41  42  43  44  45  46  47  48  49  50  51  52  53  54  55  56  57  58  59  60  61  62  63  64  65  66  67  68  69  70  71  72  73  74  75  76  77  78  79  80  81  82  83  84  85  86  87  88  89  90 | Precental gyrus  Precental gyrus  Superior frontal gyrus, dorsolateral  Superior frontal gyrus, dorsolateral  Superior frontal gyrus, orbital part  Superior frontal gyrus, orbital part  Middle frontal gyrus  Middle frontal gyrus  Middle frontal gyrus, orbital part  Middle frontal gyrus, orbital part  Inferior frontal gyrus, opercular part  Inferior frontal gyrus, opercular part  Inferior frontal gyrus, triangular part  Inferior frontal gyrus, triangular part  Inferior frontal gyrus, orbital part  Inferior frontal gyrus, orbital part  Rolandic operculum  Rolandic operculum  Supplementary motor area  Supplementary motor area  Olfactory cortex  Olfactory cortex  Superior frontal gyrus, medial  Superior frontal gyrus, medial  Superior frontal gyrus, medial orbital  Superior frontal gyrus, medial orbital  Gyrus rectus  Gyrus rectus  Insula  Insula  Anterior cingulate and paracingulate gyri  Anterior cingulate and paracingulate gyri  Median cingulate and paracingulate gyri  Median cingulate and paracingulate gyri  Posterior cingulate gyrus  Posterior cingulate gyrus  Hippocampus  Hippocampus  Parahippocampal gyrus  Parahippocampal gyrus  Amygdala  Amygdala  Calcarine fissure and surrounding cortex  Calcarine fissure and surrounding cortex  Cuneus  Cuneus  Lingual gyrus  Lingual gyrus  Superior occipital gyrus  Superior occipital gyrus  Middle occipital gyrus  Middle occipital gyrus  Inferior occipital gyrus  Inferior occipital gyrus  Fusiform gyrus  Fusiform gyrus  Postcentral gyrus  Postcentral gyrus  Superior parietal gyrus  Superior parietal gyrus  Inferior parietal, but supramarginal and angular gyri  Inferior parietal, but supramarginal and angular gyri  Supramarginal gyrus  Supramarginal gyrus  Angular gyrus  Angular gyrus  Precuneus  Precuneus  Paracentral lobule  Paracentral lobule  Caudate nucleus  Caudate nucleus  Lenticular nucleus, putamen  Lenticular nucleus, putamen  Lenticular nucleus, pallidum  Lenticular nucleus, pallidum  Thalamus  Thalamus  Heschl gyrus  Heschl gyrus  Superior temporal gyrus  Superior temporal gyrus  Temporal pole: superior temporal gyrus  Temporal pole: superior temporal gyrus  Middle temporal gyrus  Middle temporal gyrus  Temporal pole: middle temporal gyrus  Temporal pole: middle temporal gyrus  Inferior temporal gyrus  Inferior temporal gyrus | PreCG  PreCG  SFG  SFG  SFGorb  SFGorb  MFG  MFG  MFGorb  MFGorb  IFGoper  IFGoper  IFGtri  IFGtri  IFGorb  IFGorb  ROL  ROL  SMA  SMA  OLF  OLF  SFGmed  SFGmed  SFGmorb  SFGmorb  REC  REC  INS  INS  ACG  ACG  MCG  MCG  PCG  PCG  HIP  HIP  PHG  PHG  AMYG  AMYG  CAL  CAL  CUN  CUN  LING  LING  SOG  SOG  MOG  MOG  IOG  IOG  FFG  FFG  PoCG  PoCG  SPG  SPG  IPL  IPL  SMG  SMG  ANG  ANG  PCUN  PCUN  PCL  PCL  CAU  CAU  PUT  PUT  PAL  PAL  THA  THA  HES  HES  STG  STG  TPOsup  TPOsup  MTG  MTG  TPOmid  TPOmid  ITG  ITG | L  R  L  R  L  R  L  R  L  R  L  R  L  R  L  R  L  R  L  R  L  R  L  R  L  R  L  R  L  R  L  R  L  R  L  R  L  R  L  R  L  R  L  R  L  R  L  R  L  R  L  R  L  R  L  R  L  R  L  R  L  R  L  R  L  R  L  R  L  R  L  R  L  R  L  R  L  R  L  R  L  R  L  R  L  R  L  R  L  R |
